# Inequalities in the prevalence, diagnosis awareness, treatment coverage and effective control of diabetes: a small area estimation analysis in Iran

**DOI:** 10.1186/s12902-023-01271-z

**Published:** 2023-01-18

**Authors:** Lida Perseh, Maryam Peimani, Erfan Ghasemi, Ensieh Nasli-Esfahani, Negar Rezaei, Farshad Farzadfar, Bagher Larijani

**Affiliations:** 1 Non-Communicable Diseases Research Center, Endocrinology and Metabolism Population Sciences Institute, Tehran University of Medical Sciences, Tehran, Iran; 2 Diabetes Research Center, Endocrinology and Metabolism Clinical Sciences Institute, Tehran University of Medical Sciences, Tehran, Iran; 3 Endocrinology and Metabolism Research Center, Endocrinology and Metabolism Clinical Sciences Institute, Tehran University of Medical Sciences, Tehran, Iran

**Keywords:** Inequality, Diabetes mellitus, Spatial variation, Prevalence, Awareness of diagnosis, Treatment coverage, Effective control, Iran

## Abstract

**Objective:**

This study aims to assess geographic inequalities in the prevalence, awareness of diagnosis, treatment coverage and effective control of diabetes in 429 districts of Iran.

**Methods:**

A modelling study by the small area estimation method, based on a nationwide cross-sectional survey, Iran STEPwise approach to surveillance (STEPS) 2016, was performed. The modelling estimated the prevalence, awareness of diagnosis, treatment coverage, and effective control of diabetes in all 429 districts of Iran based on data from available districts. The modelling results were provided in different geographical and socio-economic scales to make the comparison possible across the country.

**Results:**

In 2016, the prevalence of diabetes ranged from 3.2 to 19.8% for women and 2.4 to 19.1% for men. The *awareness of diagnosis ranged from 51.9 to 95.7% for women and* 35.7 to 100% for men. The *rate of treatment coverage ranged from 37.2 to 85.6% for women and 24.4 to 80.5% for men. The* rate of effective control *ranged from 12.1 to 63.6% for women and 12 to 73% for men.* The highest treatment coverage rates belonged to Ardebil for women and Shahr-e-kord for men. The highest effective control rates belonged to Sanandaj for women and Nehbandan for men. Across Iran districts, there were considerable differences between the highest and lowest rates of prevalence, diagnosis awareness, treatment coverage, and effective control of diabetes. The concentration indices of diabetes prevalence, awareness of diagnosis, and treatment coverage were positive and significant for both sexes.

**Conclusion:**

Findings of this study highlight the existence of inequalities in diagnosis awareness, treatment coverage, and effective control of diabetes in all Iran regions. More suitable population-wide strategies and policies are warranted to handle these inequalities in Iran.

## Introduction

Diabetes is one of contemporary time’s most challenging and demanding chronic diseases for global healthcare systems. The global burden of disability-adjusted life-years attributable to diabetes and elevated fasting glucose has more than doubled from 1990 to 2019 [1]. In the Middle East and North Africa (MENA) region, the average prevalence of diabetes in the adult population was 12.2% in 2019, which is the highest prevalence compared to other International Diabetes Federation (IDF) regions. Iran has the third-largest number of adults with diabetes in the IDF MENA region [2]. *Diabetes affects all socio*economic groups in Iran and even has been observed for some conditions the burden is higher in the lower socio-economic groups [3–5].

The Government of Iran has initiated a response to this growing burden with the *National* Diabetes Prevention and Control Program, the National Action Plan for Non-communicable diseases Prevention and Control, and the Iranian National Diabetes Framework [6–8]. However, besides them, achieving equality has always been one of the crucial goals of national programs; and it has garnered a special place in national health strategic plans in recent decades [9]. Iran’s geographic disparities in healthcare are vast [10–12], and therefore, *better* surveillance is needed to identify geographic patterns of, and inequalities in diabetes and its care, especially when the Sustainable Development Goal 10 of reducing inequalities within countries is to be attained. In other words, tracking inequalities in care received across diabetes populations from different geographic regions, and attempting to explain the factors affecting them are crucial to the healthcare system to boost its performance in the management of diabetes at sub-national and national levels [13].

In this regard, a study conducted in China confirms a health inequality in diabetes prevalence from 2011 to 2015 that favors the rich [14]. Another study on healthcare inequalities in diabetes confirms the inability to access diabetes management technologies, with a tenfold difference in insulin pump use by type 1 diabetes patients across specialist centers in the UK [15]. However, tackling inequalities in diabetes care necessitates an understanding of current disparities throughout the entire spectrum of diabetes care– from early diagnosis to effective treatment and control [16]. Thus, this study aimed to assess geographic inequalities in the prevalence, awareness of diagnosis, treatment coverage, and effective control of diabetes in 429 districts of Iran using recent district-specific aggregate data. Our results can facilitate prioritizing resources and evidence-based targeting of programs and policies to address the identified disparities at sub-national and national levels.

## Methods

### Data source and study population

This study used publicly available data (https://vizit.report/panel/districtSteps/en/main.html#/ forest Location) from the Iran 2016 STEPS survey, implemented by the Ministry of Health and Medical Education with the support of the World Health Organization [17]. A cluster random sampling frame was considered for proportional-to-size sampling to recruit candidates from Iran’s 30 provinces, including both urban and rural regions within each province. A total of 30,541 individuals participated in this study. It should be noted that Iran is composed of 31 provinces and 429 districts. The data from the STEPS survey was collected at the provincial level and reported at the individual level. Also, participants in that study were from 389 (out of 429) districts in Iran, and data for the remaining 40 districts was not available. To meet this gap, we estimated the prevalence of diabetes, awareness of diagnosis, treatment coverage, and effective control for all districts of Iran, and the provided data in this study is aggregated, not individual.

### Small area estimation method

The STEPS surveys generate a range of invaluable data for monitoring within the country level (at the national and provincial levels). However, provincial-level data do not adequately capture the extent of geographical inequalities, which restricts the scope for evaluating progress locally within and between district units. But, the national surveys data cannot directly utilize to produce reliable disaggregate level estimates due to the very small sample sizes. In the literature, an area is regarded as small if the area-specific sample is not large enough to support a direct survey estimator of adequate precision with an unacceptably large coefficient of variation. One alternative way to this issue is to use small area estimation (SAE) techniques. The SAE approach provides reliable estimates for such small areas with small sample sizes by borrowing strength from data of other areas. The SAE techniques are based on model-based survey estimation methods. The SAE method uses indirect small area estimators that make use of the sample data from related areas through linking models, and thus increases the effective sample size in the small areas. Such estimators can produce considerably smaller variation coefficient than direct estimators, on condition that the linking models are valid [18]. Generalized linear mixed models are the basis of many SAE methods that takes into account *auxiliary information* from different sources to improve the precision of *direct* small area *estimates. A linear regression model with spatially correlated errors can be used, where small area data are spatially dependent* [19]*. In this study, we used the Bayesian spatial hierarchical regression model* at both provincial and district levels for different variables.

To utilize the neighborhoods information in the spatial analysis of small areas, the neighborhood matrix was used in such a way that the districts that were neighbors and shared a border were given a weight of 1 and the other components of the matrix were given a weight of 0. This neighborhood matrix was used in conditional auto-regressive normal distribution in spatial random effects. Using the considered prior distribution and the Likelihood distribution of the data, the posterior distribution was obtained, and by taking a sample of the posterior distribution of the original model and considering the means and 2.5 and 97.5 percentiles, a 95% uncertainty interval was calculated for each output at the district and gender levels. The 95% uncertainty intervals were used to examine the differences between districts; districts that their uncertainty intervals intersect each other for a particular indicator (e.g., diabetes prevalence), they would not differ with one another significantly. Due to the limitation in sample sizes for age *disaggregation* across the districts of the country (for each sex) and the lack of sufficient accuracy in estimating the prevalence and mean based on the age disaggregation, the estimates were calculated only at all-age levels.

Small area estimation modelling *was performed using the* R2Win BUGS package in R and OpenBUGS software. Visualization was done by R for Windows version 3.6.1. Statistical analyses were carried out by STATA version 11.

### Study variables

The variables used for the study are the prevalence, awareness of diagnosis, treatment coverage and effective control of diabetes. Diabetes prevalence was defined by self-reported use of a glucose lowering medication or biochemical evidence of diabetes using the WHO definition: fasting plasma glucose (FPG) of 126 mg/dl or higher [20]. Awareness of diagnosis was defined by two questions. Respondents were asked: whether or not they had ever had a blood glucose test, and whether or not they had ever been diagnosed with diabetes by any healthcare provider. Among those who had been tested, we then quantified the percentage of all patients with diabetes who reported having been diagnosed with diabetes as a measure of awareness of diagnosis. We defined treatment coverage as currently taking diabetes medications. In this regard, among those who had been tested and diagnosed (being aware of diagnosis), we calculated the percentage of the population who received medications for diabetes. Finally, among those receiving medications, we determined whether or not their diabetes was in control as a measure of effective control of diabetes. Effective control was defined as HbA1c < 8.0%.

We used an asset-based approach to estimate wealth using a multivariate statistical technique known as principal component analysis (PCA) [21]. This method which is frequently adopted in larger data sets, involves the use of asset-based indices and housing characteristics to create a wealth index indicating long-run economic status. We used data from the survey of household expenses and income in 2017 [22].

### Inequality measurement

To assess geographical inequalities in the prevalence, awareness of diagnosis, treatment coverage and effective control of diabetes across socio-economic groups, this study followed the methodology of Wagstaff et al. [23]. This includes visualization and estimation of inequality using the concentration curve (CC) and the concentration index (CI). The CC plots the cumulative percentage of health variables on the vertical axis, against the cumulative share of the population (ranked from the lowest to the highest by an indicator of socio-economic status) on the horizontal axis. The CC above (below) the line of equality indicates greater (lesser) health among the rich (poor). The CI is defined as twice the area between the CC and the line of equality (the 45-degree line), ranging from − 1 to + 1. When there is no socioeconomic-related inequality, the CI is zero. The concentration index can be computed for good health as well as ill health. The index has a value less than 0 when the curve lies above the equality line, indicating disproportionate concentration of the health variable among the poor, and a value greater than 0 when it lies below the equality line, indicating disproportionate concentration of the health variable among the rich. Note that, since the CI in the survey of household expenses and income 2017 was calculated at the regional level, the geographical term was used to determine inequalities.

We also calculated the extreme ratio for each of the variables, which is the highest value divided by the lowest value showing the variation between the extremes.

## Results

### Prevalence of diabetes in districts

**Fig. 1:**
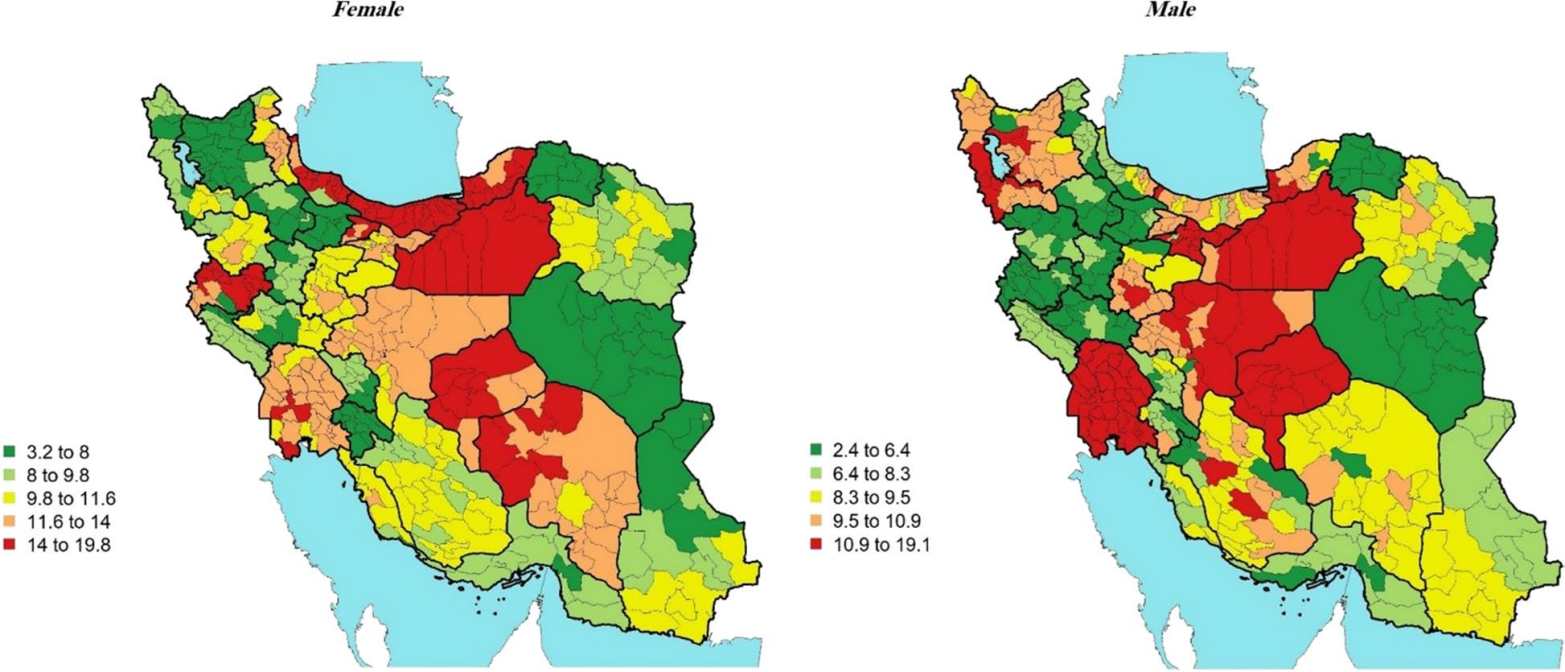
Prevalence of diabetes (%)

The results are presented separately for men and women at the districts level. In 2016, all-age prevalence of diabetes for Iranian women was 11.55% (95% UI: 10.8, 12.3%) which was slightly higher than among men, 10.01% (9.23, 10.78%) (Table 1). Across Iran districts, prevalence of diabetes ranged from 3.2 to 19.8% for women (i.e., a 6-fold difference between the highest rate and the lowest one), and 2.4 to 19.1% for men (i.e., an 8-fold difference between the highest and the lowest rates) (Fig. 1). The highest prevalence of diabetes in women–19.84% (9.28, 31.53%) was among people from the western part of Iran, Kermanshah district, and the highest prevalence in men–19.10% (10.56, 28.11%) was among people from the most central part of Iran, Yazd district. Likewise, the lowest prevalence of diabetes in women–3.18% (0.0, 8.39%) was among people from a district in north west part of Iran, Marand, East Azerbaijan Province. For men, the lowest prevalence 2.36% (0.0, 6.82%) was seen in a district in *north east part of Iran,* Maneh and Samalqan, North Khorasan Province.

**Table 1:** Lowest and highest rates of each of the variables in 429 districts by sex

Variables	Sex	Min	Max	Range
Prevalence of diabetes	Female	10.79	12.3	1.51
Prevalence of diabetes	Male	9.23	10.8	1.57
Awareness of diagnosis	Female	75	80.49	5.49
Awareness of diagnosis	Male	66.63	74.03	7.4
Treatment coverage	Female	56.19	62.97	6.78
Treatment coverage	Male	48.93	57.3	8.37
Effective control	Female	31.58	40.45	8.87
Effective control	Male	30.73	41.14	10.41

### *Awareness of diagnosis* in districts

**Fig. 2:**
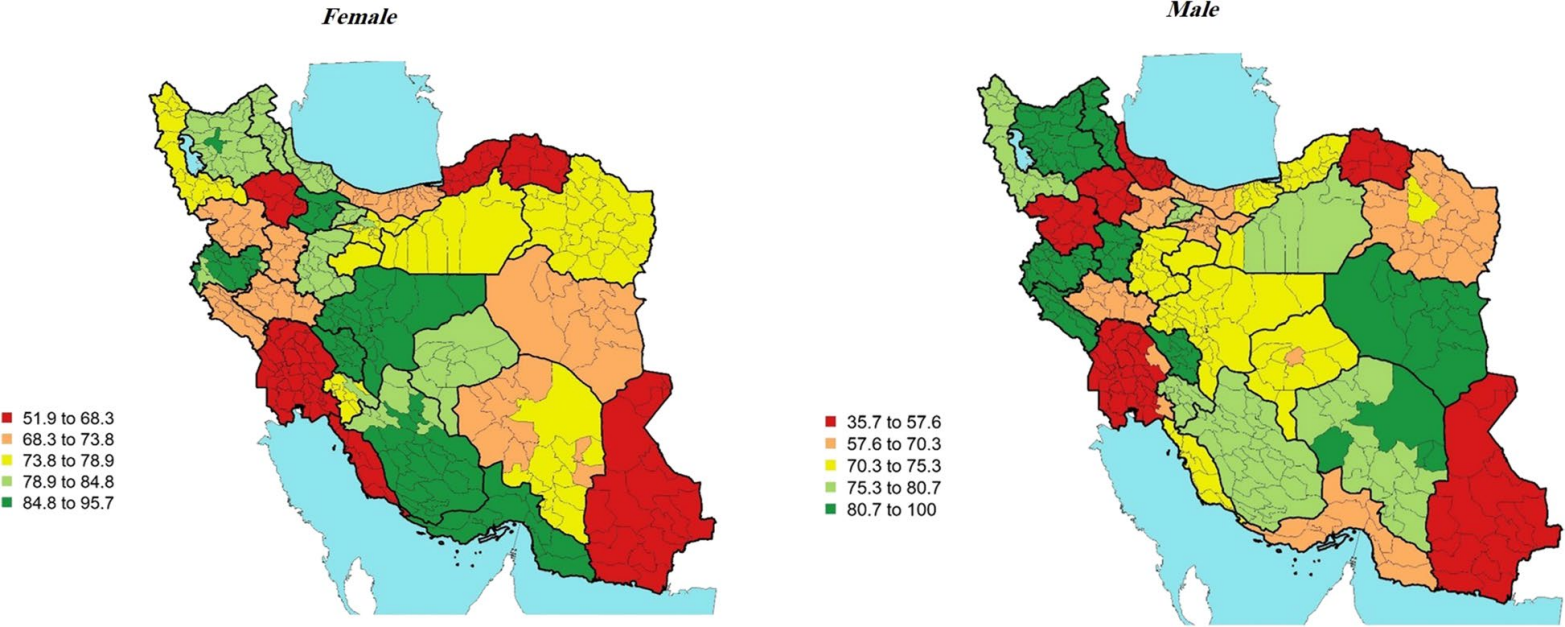
Awareness of diagnosis (%)

In terms of being aware of diabetes diagnosis in 2016, the all-age awareness of diagnosis for women and men was *77.74%* (75.0, 80.49%) and 70.33% (66.63,74.03%) respectively (Table 1). Across Iran districts, the highest rate of awareness of diagnosis in women–95.7% (80.78, 100%) in the districts of Qazvin province–was almost two times as high as the lowest–51.9% (38.19, 64.98%) in the districts of Sistan and Balouchestan province. Moreover, the highest rate of awareness of diagnosis in men–100% (71.25, 100%) in the districts of South Khorasan province–was almost three times as high as the lowest–35.7% (24.84, 46.43%) in the districts of North Khorasan (Fig. 2).

### Rate of *treatment coverage* in districts

**Fig. 3:**
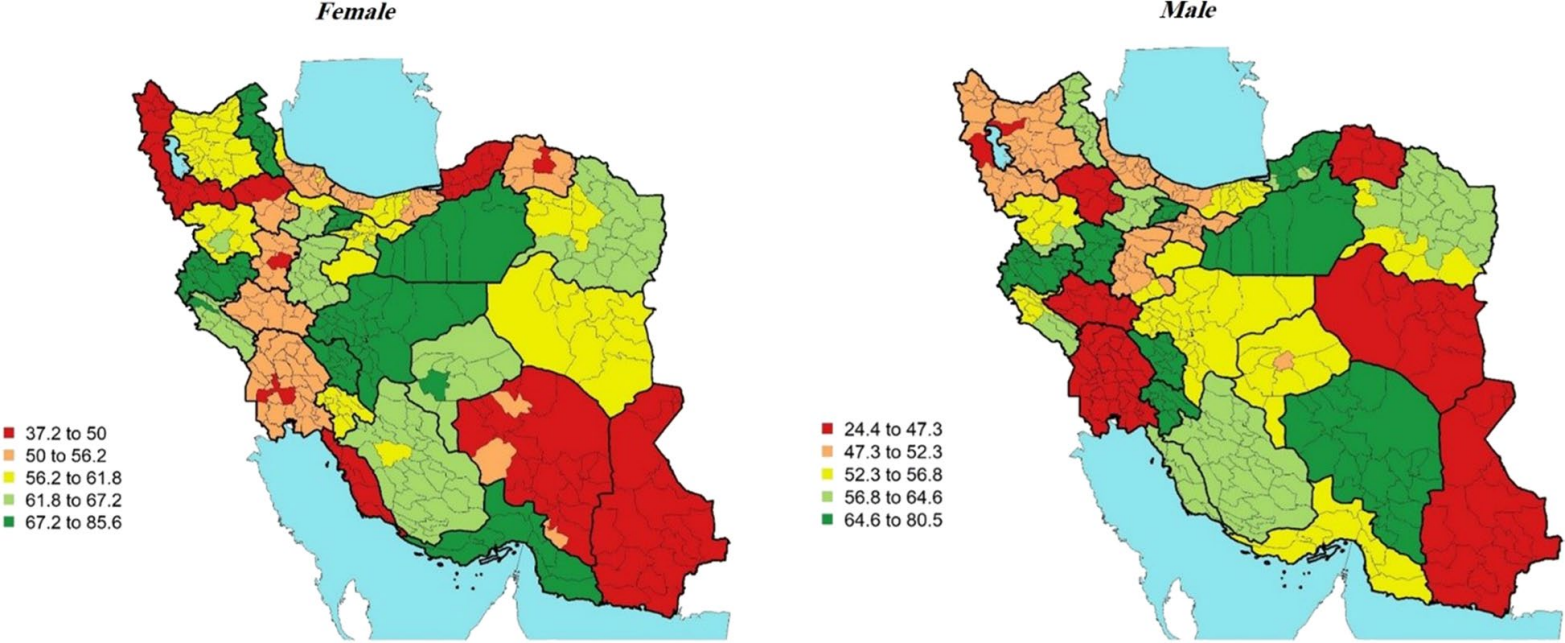
Treatment coverage (%)

In terms of diabetes treatment coverage in 2016, the all-age rate of treatment coverage for women and men was *59.58%* (56.16, 62.97%) and 53.11% (48.93,57.3%) respectively (Table 1). The rate of treatment coverage in women across districts varied from the highest rate of 85.6% (58.31, 100%) in Ardebil to that of the lowest rate of 37.2% (14.70, 57.73%) in Gorgan, indicating a > 2-fold difference between the highest and the lowest ones. The rate of treatment coverage in men across districts also varied from the highest rate of 80.5% (57.06, 100%) in Shahr-e-Kord to that of the lowest rate of 24.4% (11.24, 35.43%) in Zanjan, indicating a > 3-fold difference between them (Fig. 3).

### Rate of effective control in districts

**Fig. 4:**
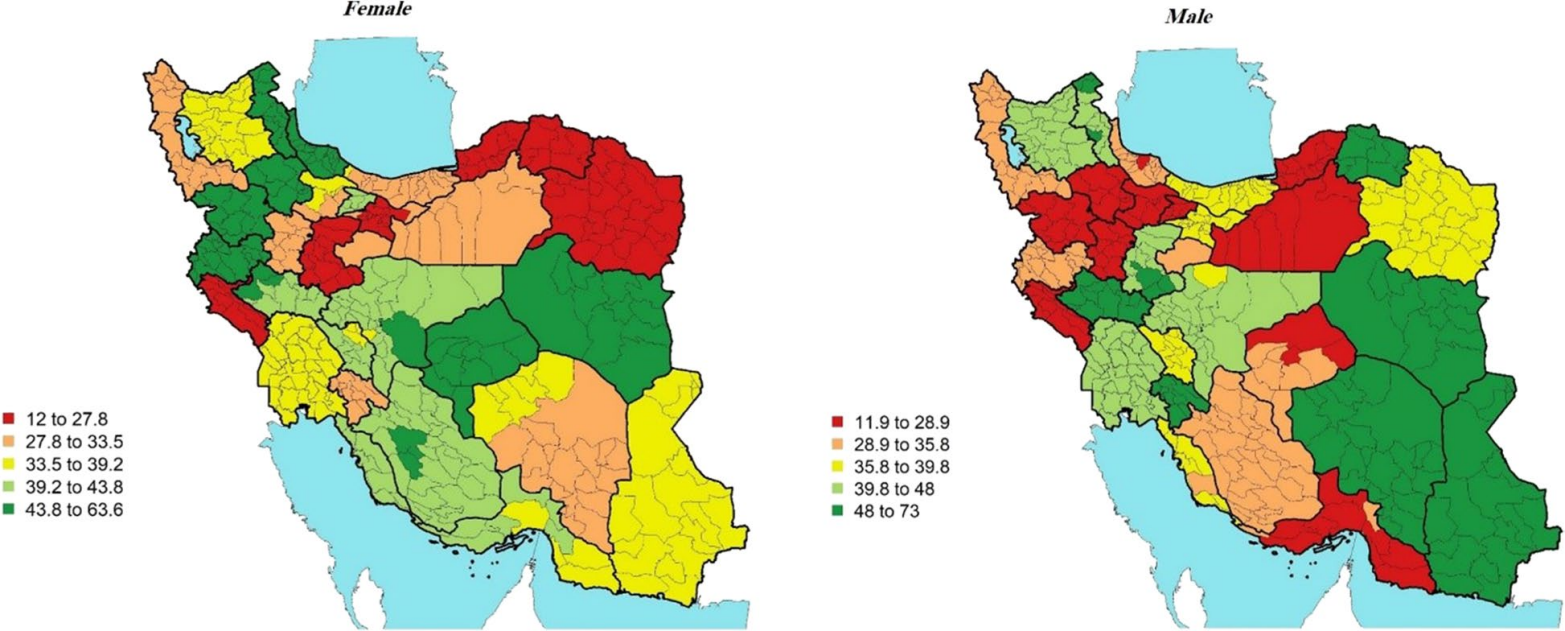
Effective control (%)

In terms of effective control of diabetes, in 2016, the all-age rate of effective control for Iranian women and men was *36.01%* (31.58, 40.45%) and *35.93%* (30.73, 41.14%) respectively (Table 1). The difference between the highest and lowest rates was considerable for both sexes across Iran districts as well. The highest rate of effective control of diabetes in women–63.6% (34.72, 95.20%) in Sanandaj district was five times as high as the lowest–12.1% (2.52, 21.32%) in Arak district. Moreover, the highest rate of effective control in men–73% (20.16, 100%) in Nehbandan district was six times as high as the lowest–12% (2.75, 20.70%) in Divandarreh district (Fig. 4).

### Inequalities in prevalence, awareness of diagnosis, treatment coverage and effective control of diabetes

Sex-stratified concentration index values for the prevalence, awareness of diagnosis, treatment coverage and effective control of diabetes are reported in Table 2. As shown in this table, the concentration indices of diabetes prevalence for both sexes were positive and significant, indicating that the rich are at a higher risk of having diabetes. Concentration indices of awareness of diagnosis for men (0.014, 95% UI: 0.004─0.024) and women (0.03, 95% UI: 0.022─0.037) were also positive and significant. However, women had a larger concentration index than men (i.e., 0.03 vs. 0.01), suggesting a stronger disparity in being aware of diabetes diagnosis across socioeconomic levels in women. The same results were observed for concentration indices of diabetes treatment coverage in both sexes (Table 2). Concentration indices of treatment coverage were positive and significant for men and women, and again, women had a larger concentration index than men (i.e., 0.03 vs. 0.01), indicating greater inequality in women regarding treatment coverage. Concentration indices for effective control in both sexes were negative and significant meaning that control variable was more concentrated among patients of lower income groups.

**Table 2:** Sex-stratified concentration indices for the prevalence, awareness of diagnosis, treatment coverage and effective control of diabetes

	Women		Men	
	Estimate	95% UI	Estimate	95% UI
Prevalence of diabetes	0.03^*^	(0.014, 0.045)	0.02^*^	(0.004, 0.036)
Awareness of diabetes diagnosis	0.03^*^	(0.022, 0.037)	0.014^*^	(0.004, 0.024)
Coverage of diabetes treatment	0.03^*^	(0.019, 0.038)	0.012^*^	(0.001, 0.023)
Effective control of diabetes	−0.015^*^	(−0.029,–0.002)	−0.043^*^	(− 0.06, − 0.026)

Note. 95% uncertainty intervals are in parentheses

^*^All *P*-values are less than 0.01

## Discussion

This study analyzed sex- and geographic-related inequalities in the prevalence, awareness of diagnosis, treatment coverage, and effective control of diabetes within 429 districts of Iran. The study revealed that, generally, *there exist inequalities in* the prevalence, awareness of diagnosis, treatment coverage, and effective control of diabetes in all districts across Iran. SDG Target 17.18 specifically calls for countries to increase the availability of data disaggregated by all relevant inequality dimensions, such as sex, age, income/wealth, education, geographic location, or other characteristics, to identify and track disadvantaged populations within countries [24].

In this study, the analysis of diabetes prevalence by sex showed that women had a higher diabetes prevalence rate when compared with men (11.55% versus 10.01%), and the confidence intervals around these prevalence estimates did not overlap. Based on this finding, it is likely that significant differences in diabetes prevalence do exist between women and men across districts in Iran. This finding, consistent with some previous studies [25–27], may be related to the higher prevalence of abdominal obesity in women [28]. Moreover, there was a multi-fold difference between the highest and lowest prevalence levels across districts, indicating a wide disparity in diabetes prevalence by geographic areas in the country. Findings from the Prospective Epidemiological Research Studies in IrAN (PERSIAN) Cohort on non-communicable diseases [29] that started in 2014 in 19 centers, intending to include all the major ethnic groups in various regions of Iran, confirm this disparity in the prevalence of diabetes across the different geographical areas of the country. This high disparity in the prevalence of diabetes among different regions necessitates the importance of thorough intervention at regional and subregional levels to tackle the existing problem.

Our analysis demonstrated a *high rate of awareness of diagnosis (77.74% in women* and 70.33% in men), a moderate rate of treatment coverage (*59.58% in women* and 53.11% in men), and a low rate of effective control (36.01% in women and 35.93% in men). In addition, *women had relatively higher percentages of awareness of* diagnosis and treatment coverage than men, except for effective control, where the rates were almost the same for both sexes. Results from a cross-sectional study on nationally representative data from 28 low- and middle-income countries were similar to ours. They showed that although people with diabetes who live in middle- and upper-middle-income countries like Iran are more likely to be tested, aware of the diagnosis, and treated for their diabetes than those in low- and lower-middle-income countries, only 16–25% ultimately achieve control [30]. *A point that emerges from the current* analysis is that our health system may have problems translating services delivered into effective disease control [31]. These problems might be owing to demand-side factors, such as lack of patient engagement, inability to afford care, and sociocultural barriers; and/or supply-side factors, such as lack of services, low responsiveness of the services provided, and geographic inaccessibility [32].

*Another thought-provoking finding of this study* was related to *the extent of geographic variation for awareness of diagnosis,* treatment coverage, and effective control of diabetes across districts *in the country.* The results revealed that considerable differences still exist between the highest and lowest estimates across districts in Iran. These differences in diagnosis awareness, treatment coverage, or effective control can also explain the observed geographic disparity in diabetes prevalence. However, these multi-fold differences between the maximum and minimum percentages across districts may show disparities in the processes of care and health outcomes relevant to diabetes management [33, 34] and also show the need to close gaps in diabetes care. In this regard, diabetes prevention and management must be prioritized as a national agenda by policymakers, with regional progress closely monitored [7]. Building better healthcare infrastructure, improving standardized treatment services, implementing coordinated multilevel interventions to reduce geographic disparities in care, and using digital technology such as virtual clinics could help to increase awareness, treatment, and control of diabetes [7, 8, 29]. Moreover, an adopted national clinical practice guideline that considers limited local resources and some problems in accessing Iranian patients’ anti-diabetic drugs, especially modern treatments due to sanctions, should be prepared and regularly updated [35, 36].

Using the concentration index approach, we quantified *the magnitude* of income-related inequalities in prevalence, diagnosis *awareness,* treatment coverage, and effective control. The findings of this study indicated that the risk of diabetes prevalence for both sexes was significantly higher among people with higher socioeconomic status. Recent literature from high-income countries reports higher rates of diabetes in the poorer socioeconomic groups [37–39], and earlier literature from these countries reported a pattern where the rich were at higher risk of diabetes [40]. This proposes that during economic transitions, harmful health behaviors are initially encountered in the higher socioeconomic *sections* of society and are later shifted to the lower socioeconomic groups [41]. Similar events may be taking place in Iran. The pro-rich pattern of diabetes and small magnitude of the concentration index seen in the present study may suggest that the country is in an economic transitional stage.

Our results showed that diagnosis *awareness and* treatment coverage were both more concentrated among patients of higher income groups, consistent with Wang et al., [25] who found a disproportionate concentration of individuals receiving antidiabetic medication among the rich in urban and rural areas of China. Their results revealed that the increase in household per capita income significantly enhanced the likelihood of receiving antidiabetic medication. This result is consistent with *another study conducted* in Bangladesh, which showed that treatment of diabetes and hypertension was more concentrated among the wealthiest than the poorest groups [42]. The other study, conducted in South Africa, indicated that the utilization of diabetes screening services and awareness of diagnosis were more concentrated in the two wealthiest quintiles [43]. Moreover, similar results were obtained in a study about diabetes treatment coverage in 55 low- and middle-income countries, in which Iran and some other Middle Eastern countries were also included. The results of that study showed that there was a gradient of greater treatment coverage with increasing household wealth [44]. Eventually, findings from a study on socioeconomic inequalities for non-communicable diseases in Fasa, southern Iran, and one on socioeconomic inequalities in risk factors for non-communicable diseases in Kurdistan, western Iran, showed significant socioeconomic inequalities in diabetes and its risk factors in these regions [45, 46]. It all shows that public health strategies should concentrate more on socioeconomically disadvantaged people as a whole.

The results, in addition to showing low rates of diabetes effective control in both sexes, also indicated that effective control was more concentrated among the poor (negative concentration index). The higher level of effective control of diabetes among the patients of lower-income groups may be attributed to the implementation of the health reform plan in Iran [31, 47], which was launched in 2014 and aimed to offer equitable essential public health services for all Iranian people, especially the most vulnerable citizens. However, *it may be worth referring to* the small magnitude of the concentration indices observed in this study, which may be a reflection of the Iranian universal health care system with relatively equal access to healthcare. Universal Health Coverage (UHC) [48] is another recently implemented policy in Iran, with the goal of increasing the number and diversity of services provided to poor patients, which have played a substantial role in decreasing inequality in the country.

The limitations of this work are the complex modelling methods and possible statistical errors during *analysis that* we tried to reduce by using an appropriate model. However, this is the first district-based study of tracking inequalities in the prevalence, awareness of diagnosis, treatment coverage, and effective control of diabetes in all Iran districts, which is the greatest strength of this work. Other limitations could be probable mistakes in the study’s variables measurements of included participants that were used as the base data for the modelling, which is outside the scope of this study because no new measurements were done for modelling. Another limitation is that since this study compared groups, the results may not be applicable to individuals. As a result, they may be prone to the ecological fallacy.

## Conclusion

Findings of this study confirm the existence of inequalities in the prevalence, awareness of diagnosis, treatment coverage, and effective control of diabetes in all Iranian regions. Regarding disparities in diabetes care and health outcomes relevant to diabetes management in almost all districts of Iran, proper legislation and accurate implementation of national action plans are needed to stop these trends. Spatial variations of prevalence, awareness of diagnosis, treatment coverage, and effective control of diabetes estimated in this study provide beneficial measures for national and sub-national authorities and policymakers to handle these inequalities in Iran.

## Data Availability

The datasets generated and/or analysed during the current study are available in https://vizit.report/panel/districtSteps/en/main.html#/forestLocation.
